# Moderate-Vigorous Physical Activity, Family Support, Peer Support, and Screen Time: An Explanatory Model

**DOI:** 10.3390/ijerph192316177

**Published:** 2022-12-03

**Authors:** Daniel Sanz-Martín, José Luis Ubago-Jiménez, Germán Ruiz-Tendero, Félix Zurita-Ortega

**Affiliations:** 1 Department of Didactics of Musical, Plastic and Corporal Expression, Faculty of Humanities and Educational Sciences, University of Jaén, 23071 Jaén, Spain; 2 Department of Didactics Musical, Plastic and Corporal Expression, Faculty of Education Science, University of Granada, 18071 Granada, Spain; 3 Department of Languages, Arts and Physical Education Teaching, Faculty of Education, Complutense University of Madrid, 28040 Madrid, Spain

**Keywords:** physical activity, social support, adolescent, health

## Abstract

It is important to investigate how the different factors of physical activity and screen time influence each population group in order to design effective proposals for health promotion. This study aims to: (1) create an explanatory model to establish the relationships between moderate-vigorous physical activity time, screen time, family support, and peer support of adolescents in the region of Soria (Spain); (2) contrast the explanatory structural model according to the intensity of physical activity. A representative sample of 694 adolescents, aged 12–17 years, from the region of Soria was selected. The Four by One-Day Physical Activity Questionnaire, the Parent Support Scale, and the Peer Support Scale were administered. The data were treated according to a structural equation model to demonstrate the relationships between the study variables. The Chi-square *p*-values and standardised fit indices (CFI, NFI, IFI, TLI, and RMSEA) were appropriate. Moreover, acceptable parameters were obtained in the general equations. The theoretical model is useful to explain the relationships between moderate-vigorous physical activity, family support, peer support, and screen time. In addition, models that differentiate between moderate and vigorous physical activity independently are also useful. Peer support plays a particular role relative to physical activity time, and family support plays a specific role regarding screen time.

## 1. Introduction

Physical activity (PA) can be beneficial for the physical, psychological/social, and cognitive health of children and adolescents [1]. Some of these benefits are: a reduction in adiposity, the prevention of overweight and obesity, an improvement in cardiometabolic biomarkers, the prevention of metabolic syndrome, an improvement in bone health, the reduction of anxiety, and an improvement in psychological well-being [1,2,3]. To achieve such benefits, PA recommendations must be followed. For children and adolescents, 60 min/day of moderate-vigorous physical activity (MVPA) and at least 3 days/week of vigorous activity, as well as bone and muscle strengthening, are recommended [4]. In addition, it has also been shown that engaging in sedentary behaviours is negatively associated with health [5,6]. Therefore, it is recommended that this population group should not engage in more than 2 h/day of recreational screen time (ST) [2,4].

Despite the evidence regarding the benefits of PA practice and the harms of ST, the reality is that these levels are much lower and much higher than expected, respectively. Globally, 81% of adolescents are inactive, i.e., they do not meet the practice recommendations, although these levels vary from country to country [7]. A total of 71.1% of European adolescents are inactive [8], with 76.6% of adolescents from Spain classified as inactive [7].

In the study conducted by Serrano-Sanchez [9], 66.6% of Spanish adolescents exceeded the recommended screen time, and 34.7% spend more than 4 h/day on screens. Moreover, it is worth noting that 38.4% exceed the recommended time spent watching TV alone, without taking other activities into account. The same is true of the 15.1% who only use the computer.

Knowing precisely which factors influence PA levels in each population would help to better adapt actions to reverse this problem [10]. Khan [11] and Lawler [12] found that one of the factors influencing physical activity in adolescents is social support, with a positive and significant relationship between such support and adherence to PA recommendations. Another factor in PA could be ST, but more scientific evidence is needed to comprehensively understand this relationship [13]. Some studies have shown significant associations between ST and PA [9,14] and others have not [15,16]. This could be due to the existence of a third variable that has not been considered in previous research. This could influence the relationship between the other two, although no previous studies have been found to prove this.

Social support has been considered a fundamental variable in several models and theories that study the determinants of PA practice [17,18], so it could also directly influence the PA and ST of adolescents. For example, Noland and Feldman’s exercise behavior model [17] considers social support as one of the modifying factors of PA, and specifically, the type of aspects to be enhanced. Another example is The Youth Physical Activity Promotion Model, by Welk [18]. According to this model, family influence and peer influence are considered to be strengthening factors, i.e., their purpose is to consolidate PA behaviours.

Based on the above, the following research objectives were proposed: (1) to create an explanatory model to establish the relationships between time of MVPA, ST, family support, and peer support of adolescents in the region of Soria; (2) to contrast the explanatory structural model in terms of the intensity of PA.

## 2. Materials and Methods

### 2.1. Design and Subjects

The study design was cross-sectional, with correlational analysis of MVPA, social support for PA practice, and screen time.

The study population consisted of 3224 adolescents, aged 12–17 years, in the region of Soria (Spain). A non-probabilistic convenience sample was used. The sample was 694 young people (14.06 ± 1.27 years), which implies a precision error of 3.3%.

Soria is considered a disadvantaged area. This Spanish region has the third-lowest GDP and the smallest population (89,041 inhabitants). The population density is 8.64 inhabitants/km^2^. It consists of 183 municipalities, 171 of which comprise less than 1000 inhabitants [19].

### 2.2. Instruments and Variables

The following instruments were used: the Four by One-Day Physical Activity Questionnaire (FBODPAQ), the Parent Support Scale, and the Peer Support Scale.

The FBODPAQ was designed by Cale [20] for a British population and was validated by Soler et al. [21] for Spanish adolescents, with a reliability of α = 0.832. It has been used in other research prior to this study [22,23]. This questionnaire requests the time spent doing different activities and allows for the calculation of energy expenditure based on the allocation established in the protocol. The variable MVPA was calculated as the sum of the practice times in activities with an allocation of at least 4 METs/h, the moderate PA of those with an average of 4 METs/h, and the vigorous PA of those with an average of at least 6 METs/h. Likewise, the ST variable was assessed as the result of the items referring to “watching TV” and use of “computer, video games and internet”.

The Parent Support Scale and the Peer Support Scale were created by Prochaska et al. [24], although the Spanish version was used [25]. Each instrument requests the rating—using a Likert scale of 0–4 (0 = not at all; 4 = every day)—of five items related to perceived support for doing PA during the week prior to administration. The score for support from family and friends was calculated as the sum of the items scores for each scale. The reliability of the items in Cronbach’s alpha values ranged from 0.7 to 0.83. These instruments have been used in previous studies [22,26].

### 2.3. Procedure

Firstly, a document search was carried out on the research topic, but no research with the objective of this study was found. Thus, this study addresses the research from an innovative perspective regarding the determinants of adolescent PA.

Secondly, the research project was drafted. This was based on the principles of the Declaration of Helsinki, and it was approved by the Ethics Committee of the University of Granada (1478/CEIH/2020). In addition, permission to carry out the research was obtained from the head of the Provincial Directorate of Education of Soria and, subsequently, from the directors of the schools offering compulsory secondary education studies.

Subsequently, an informed consent form was given to the legal guardians of the selected young people. Only those students who returned the signed informed consent form to the research team participated in the research.

Finally, once the instruments administered had been collected, data analysis was carried out, and the research report was drafted based on the scientific literature.

### 2.4. Data Analysis

**Figure 1:**
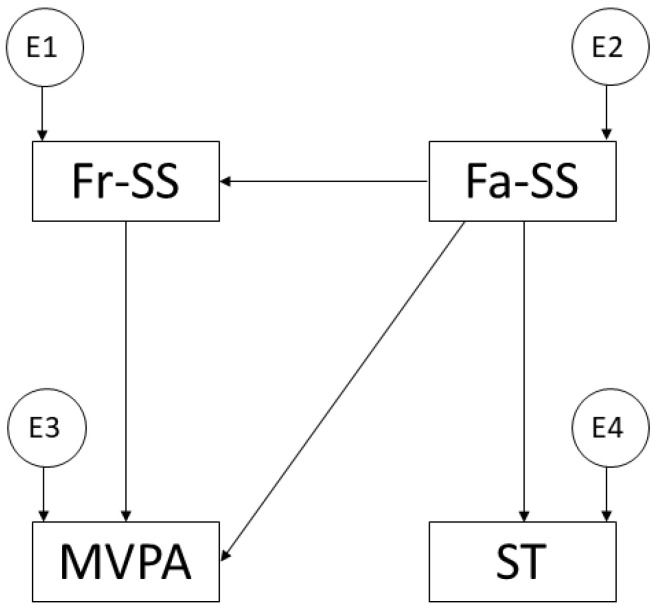
Theoretical model proposed. Note: family support (Fa-SS); friend support (Fr-SS); moderate-vigorous physical activity (MVPA); screen time (ST).

The IBM SPSS Statistics 25.0 software (IBM Corp., Armonk, NY, USA) was used for descriptive analysis to calculate the means and standard deviation of the variable measures. IBM SPSS Amos 26.0 (IBM Corp, Armonk, NY, USA) was used for the correlation analysis. This allowed for the creation of the structural equation model, which facilitated the study of the relationships between the variables that made up the theoretical model (Figure 1).

A general model was conducted for the whole sample. This was composed of a total of four observed or endogenous variables. Causal explanations between the variables were established on the basis of observed linkages, measurement reliability, and specific indicators, including measurement error. In addition, the arrows in the model represent the lines of influence between the variables, which are interpreted by the regression weights.

The fit of the proposed model was also assessed following Maydeu-Olivares [27] and McDonald and Marsh [28]. According to these authors, goodness of fit should be assessed using the Chi-square test, whereby a correct model fit is obtained based on non-significant values associated with p. Likewise, the comparative fit index (CFI) should be greater than 0.95; the normal fit index (NFI), incremental fit index (IFI) and Tucker–Lewis index (TLI) should be greater than 0.90, and the root mean square error of approximation (RMSEA) should be less than 0.1 [29,30].

## 3. Results

From the descriptive analysis, it was found that adolescents in the province of Soria performed 68.71 min/day (±46.41) of MVPA. Of this total MVPA time, 47.75 min/day (±40.18) are moderate physical activity and 21.55 min/day (±28.74) are vigorous physical activity. These young people devote 117.70 min/day (±76.69) to ST activities. In relation to perceived social support, the mean score for support from friends is 1.61 points (±0.58) and for support from family members, the mean score is 2.09 points (±0.81).

In relation to correlational analysis, the model proposed to link the variables of MVPA, family support, support from friends, and ST of the adolescent population of Soria obtained a good fit in regards to the different indicators.

The Chi-square *p*-value was significant (X2 = 11.046; df = 2; pl = 0.004). The influence of susceptibility implies that the data cannot be interpreted independently [31]. Therefore, other standardised fit indices were also calculated. The CFI value was 0.980, NFI 0.976, IFI 0.980, TLI 0.940, and RMSEA 0.081.

**Figure 2:**
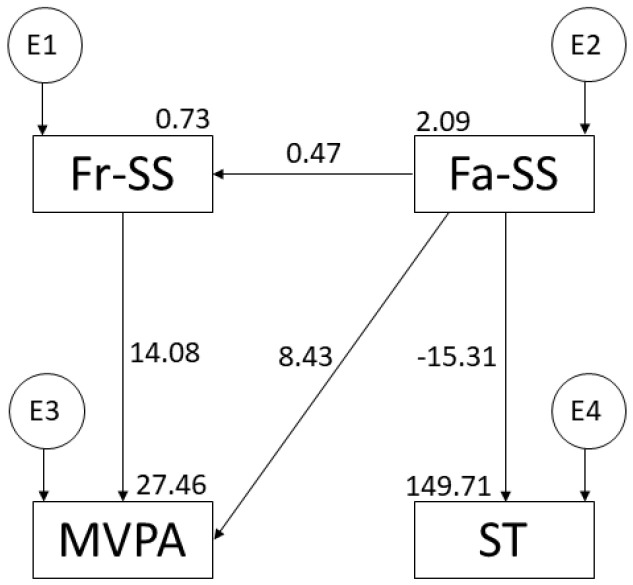
Model suggested for adolescent population. Note: family support (Fa-SS); friend support (Fr-SS); moderate-vigorous physical activity (MVPA); screen time (ST).

Figure 2 and Table 1 include the regression weights of the theoretical model regarding MVPA. The relationship between family support and friend support is positive (r = 0.474; *p* ≤ 0.001). Similarly, the relationships between peer support and MVPA (r = 14.077; *p* ≤ 0.001) and family support and MVPA (r = 8.428; *p* ≤ 0.05) are also positive. In contrast, the relationship between family support and ST is negative (r = −15.310; *p* ≤ 0.001).

**Figure 3:**
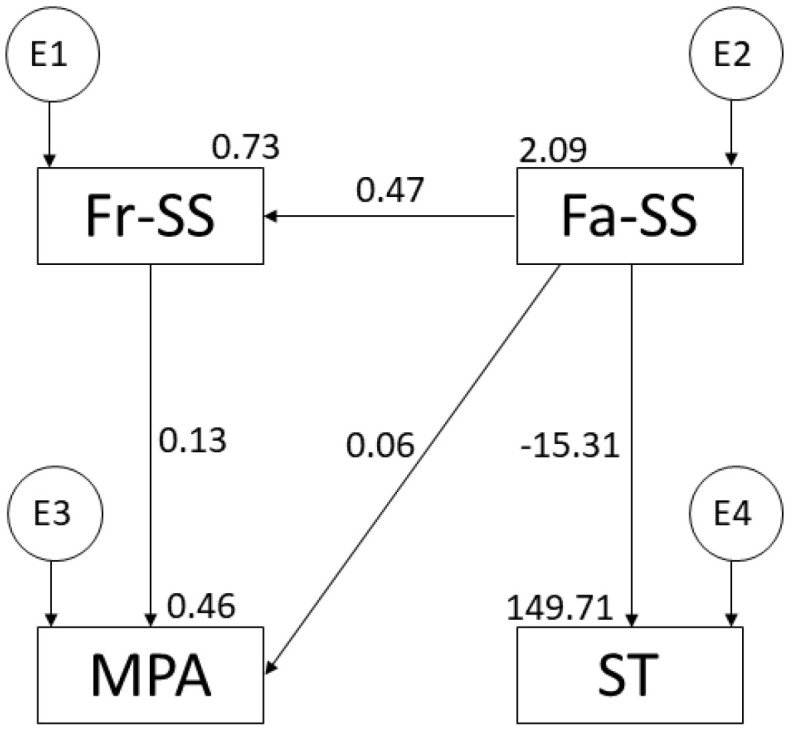
Model suggested for adolescent moderate-PA. Note: family support (Fa-SS); friend support (Fr-SS); moderate physical activity (MPA); screen time (ST).

**Figure 4:**
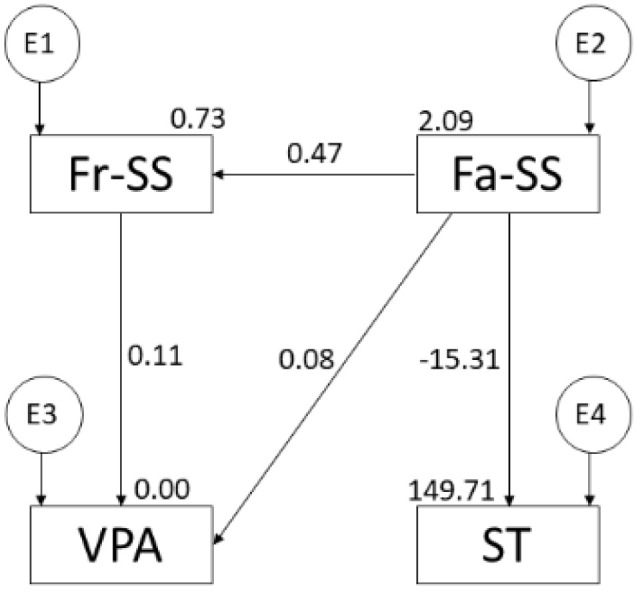
Model suggested for adolescent vigorous-PA. Note: family support (Fa-SS); friend support (Fr-SS); vigorous physical activity (VPA); screen time (ST).

The model has also been tested as a function of PA intensity, by differentiating between moderate (Figure 3) and vigorous (Figure 4) physical activity. In addition, the regression weights of these models are shown in Table 1.

The *p*-value of the moderate PA Chi-square model was non-significant (X2 = 4.281; df = 2; pl = 0.118). Although the data can be interpreted independently [31], the values of the standardised fit indices were also calculated: CFI = 0.994, NFI = 0.990, IFI = 0.994, TLI = 0.983, and RMSEA = 0.041. Furthermore, it can be seen in Figure 3 and Table 1 that there are positive relationships between family support and friend support (r = 0.474; *p* ≤ 0.001), between friend support and moderate PA (r = 0.127; *p* ≤ 0.05), and between family support and moderate PA (r = 0.058; *p* > 0.05). Likewise, the relationship between family support and ST is negative (r = −15.310; *p* ≤ 0.001).

Regarding the vigorous PA model, the Chi-square *p*-value was significant (X2 = 5.984; df = 2; pl = 0.05). In this instance, the data cannot be interpreted independently [31]. The standardised fit indices obtained values of: CFI = 0.991, NFI = 0.990, IFI = 0.991, TLI = 0.972, and RMSEA = 0.054. In addition, there are positive relationships between family support and friend support (r = 0.474; *p* ≤ 0.001), between friend support and vigorous PA (r = 0.108; *p* ≤ 0.01), and between family support and vigorous PA (r = 0.082; *p* ≤ 0.01). In contrast, there is a negative relationship between family support and ST (r = −15.310; *p* ≤ 0.001) (Figure 4 and Table 1).

## 4. Discussion

The study aims were: (1) to create an explanatory model to establish the relationships between time of MVPA, ST, family support, and peer support of adolescents in the region of Soria; (2) to contrast the explanatory structural model in terms of the intensity of PA. The results are in line with what is established in the scientific literature regarding the explanatory structural model [27,28,29,30,31]. Based on the results obtained, it can be affirmed that the theoretical model is valid to explain the relationship between the study variables (study aim 1). Moreover, this model also serves to explain these relationships by separately differentiating the intensity of PA into moderate and vigorous, (study aim 2). Next, the results will be discussed within the framework of the existing scientific literature.

In this study, a positive and significant relationship was found between young people’s daily MVPA time and the score regarding the support they perceive from friends for this practice. This relationship also holds in the moderate PA and vigorous PA models. This result is in line with the findings of Mendonça et al. [32], who conducted a systematic review and found 42 studies in which there was a positive and significant relationship between these variables, 17 in which the relationship was not significant, and none with a negative relationship.

Similar trends were also demonstrated in studies subsequent to that of Mendonça et al. Khan et al. [11] found a positive relationship between achieving recommended PA levels and peer support. Furthermore, 19.6% of adolescents with high peer support met PA recommendations, compared to 12.1% of adolescents with low peer support.

Young et al. [33] also found a positive and significant association between MVPA and social support from friends in a longitudinal study of females with measurements at 14, 17, and 23 years of age. Similarly, Morrissey et al. [34] found a positive relationship between MVPA and friend support in a longitudinal study of Iowa adolescents measured at 13, 15, and 17 years of age.

Haidar et al. [35] not only demonstrated a positive relationship between moderate PA and peer support in 8th and 11th grade adolescents of Texas (U.S.A.), but the relationship was also positive for vigorous PA.

In this study, with students from Soria, a positive and significant relationship was also found between MVPA time and family support. This relationship is maintained in the vigorous PA model, but not in the moderate PA model. This is also in line with the results of previous studies, although the predominance is not as evident in relation to friends. In the systematic review by Mendonça et al. [32], they found 21 studies in which there was a positive and significant relationship between young people’s MVPA and family support, 2 in which the relationship was negative, and 11 in which there was no significant relationship.

Khan et al. [11] demonstrated a positive relationship between achieving recommended PA levels and parental support. Furthermore, 19.1% of students with high levels of parental support met the PA recommendations. Similarly, Engels et al. [36] found a positive and significant correlation between MVPA time and family support. Haidar et al. [35] also found a positive and significant relationship between parental support and moderate PA, as well as with vigorous PA.

In this study, the importance of social support for adolescents’ PA practice is evident. The importance of the support of friends compared to that of family members is even more noteworthy. Furthermore, one of the main novelties is that there is a positive relationship between the support of family members and the support of friends for PA.

The importance found with respect to the support of friends is related to the findings of Morrissey et al. [34]. According to these authors, between the ages of 13 and 17, peer support is higher than family support. Moreover, the levels of social support decrease as the adolescent’s age increases, where the decrease in family support is greater than the decrease in support from friends.

It could also be that the onset of adolescence is a turning point in the shift in the superiority of social support from family during childhood to friends in the adolescence. This is because the mean score on the family scale in Sanz’s study [26] was higher than the mean score on the friends scale in both the third and sixth grades of primary school.

The last relationship in the model shown is related to ST and family support for PA. This association is negative and significant. This is consistent with the findings of Costigan et al. [37].

As can be seen in the above model, family and peer support are key factors in MVPA. This importance is also seen in the models for PA intensities, with the exception of the relationship between family support and moderate PA. In addition, family support is also important with respect to ST. The importance of social support has also been demonstrated in other theoretical models on different lifestyle habits. Park et al. [38] reported the indirect effect of parental support for PA on children’s body weight mediated by PA and ST. Jekauc et al. [39] also noted the importance of social support in a model that integrated other variables, namely, perceived competence, self-esteem, enjoyment, and amount of MVPA. Likewise, Lisboa et al. [40] showed relationships between the participant’s gender, PA levels, and different types of social support from family and friends.

The study conducted has some limitations. Firstly, there is a limitation derived from the study design. As it is a cross-sectional study, the measurements were taken at a specific time. This could have varied since the administration of the instruments. Furthermore, this model serves to explain the relationships between the variables in the adolescent population of Soria, and its generalisability is therefore limited. Secondly, there is a limitation linked to the PA measurement instrument. The use of a self-administered questionnaire implies the subjectivity of the participant. Consequently, this implies lower measurement precision than that obtained using an objective instrument, such as a pedometer or accelerometer. Finally, there is a limitation regarding the calculation of screen time. This is because the questionnaire items used for this calculation ask about television viewing and the use of computers, video games, and the Internet. It is, therefore, only a partial measurement of screen time, as it does not ask about other activities, such as the use of mobile phones or smartwatches.

On the other hand, the study facilitates a reflection on the research topic, which opens certain avenues for future research. It would be possible to test whether the theoretical model could be used to explain the relationships between these variables in other population groups. It would be interesting to carry out a longitudinal study to determine the temporal evolution of the relationships between variables. Finally, it would be advisable to take into account the family, but especially peers, in order to make proposals for promoting healthy habits in adolescents, especially those related to increasing physical activity and reducing screen time.

## 5. Conclusions

In conclusion, the theoretical model presented is useful for explaining the relationships between MVPA, family support, peer support, and ST. This is a consequence of obtaining acceptable parameters in the general equations. Peer support plays a specific positive role in MVPA time. Family support plays a specific negative role in ST and a positive one in MVPA time and VPA time. Likewise, family support is associated with friend support.

## Figures and Tables

**Table 1:** Structural model of the theoretical model.

	Variable Associations	R.W.				S.R.W.
		Estimations	S.E.	C.R.	*p*	Estimations
MVPA	Fr-SS ← Fa-SS	0.474	0.022	21.932	***	0.640
	MVPA ← Fr-SS	14.077	3.658	3.848	***	0.182
	ST ← Fa-SS	−15.310	3.542	−4.323	***	−0.162
	MVPA ← Fa-SS	8.428	2.709	3.112	*	0.147
MPA	Fr-SS ← Fa-SS	0.474	0.022	21.932	***	0.640
	MVPA ← Fr-SS	0.127	0.054	2.336	*	0.114
	ST ← Fa-SS	−15.310	3.542	−4.323	***	−0.162
	MPA ← Fa-SS	0.058	0.040	1.441		0.070
VPA	Fr-SS ← Fa-SS	0.474	0.022	21.932	***	0.640
	MVPA ← Fr-SS	0.108	0.038	2.823	**	0.135
	ST ← Fa-SS	−15.310	3.542	−4.323	***	−0.162
	VPA ← Fa-SS	0.082	0.028	2.919	**	0.140

Note: regression weights (R.W.); standardised regression weights (S.R.W.); standard error (S.E.); critical ratio (C.R.); family support (Fa-SS); friend support (Fr-SS); moderate-vigorous physical activity (MVPA); moderate physical activity (MPA); vigorous physical activity (VPA); screen time (ST); *** *p* ≤ 0.001; ** *p* ≤ 0.01; * *p* ≤ 0.05.

## Data Availability

Not applicable.
